# Primary intracranial neuroendocrine tumor at the sphenoid ridge with metastasis to the spinal cord: A case report

**DOI:** 10.1097/MD.0000000000040996

**Published:** 2024-12-13

**Authors:** Siyuan Pan, Shuide Chen, Seidu A. Richard, Zhigang Lan

**Affiliations:** a Department of Neurosurgery, Xiamen Branch of West China Hospital, Sichuan University, Xiamen, P.R. China; b Department of Neurosurgery, West China Hospital, Sichuan University, Chengdu, P.R. China; c Institute of Neuroscience, Third Affiliated Hospital, Zhengzhou University, Zhengzhou, P.R. China; d Department of Biochemistry and Forensic Sciences, School of Chemical and Biochemical Sciences, C. K. Tedam University of Technology and Applied Sciences (CKT-UTAS), Navrongo, Ghana.

**Keywords:** extracranial, meningioma, metastasis, neuroendocrine tumor, sphenoid ridge, spinal cord

## Abstract

**Rationale:**

Neuroendocrine tumors (NETs) originate from neuroendocrine cells and they are depicted with both nerve cells as well as hormone-producing cells. These tumors were initially discovered in extracranial locations and central nervous system involvement is often a result of metastasis. Herein, we present a very rare case of primary intracranial neuroendocrine tumor (PINET) that masqueraded as meningioma at the sphenoid ridge with metastasis to the spinal cord in a patient without a known history of extracranial NET at the time of diagnosis.

**Patient concerns:**

A 52-year-old male presented with a 2-month history of headache and decreased vision in the left eye accompanied by inarticulation in speech for 1 month.

**Diagnoses:**

Magnetic resonance imaging of the head showed a space-occupying lesion in the left sphenoid ridge which was mistaken for meningioma.

**Interventions:**

The lesion was surgically resected and immunohistochemical evaluation revealed PINET. Postoperative positron emission tomography scan and magnetic resonance imaging of the thoracolumbar spine detected a tumor nodule in the thoracolumbar region which was a metastatic tumor. The metastatic lesion at the thoracolumbar spine was surgically resected and spinal fixation was carried out to stabilize the spine. Immunohistochemical evaluation of the spinal lesion also confirmed NET. He was further treated with several cycles of adjuvant chemotherapy and radiotherapy.

**Outcomes:**

Two years’ follow-up revealed no recurrence of the tumor and he is currently well. However, we are still following the patient because of the nature of the tumor.

**Lesson:**

PINET may be capable of metastasizing to spinal cord.

## 1. Introduction

Neuroendocrine tumors (NETs) originate from neuroendocrine cells and they are depicted with both nerve cells as well as hormone-producing cells.^[1,2]^ Thus, these tumors produce or secrete amines, peptide hormones, or may contain secretory granules.^[3,4]^ These tumors were initially discovered in extracranial locations such as the lungs, gastrointestinal tract, liver, and pancreas.^[3]^ Notably, central nervous system (CNS) involvement of the tumor most often occurs as a result of metastasis, particularly the high-grade variant. Thus, it is very rare to have the tumor originating primarily from the CNS.^[1]^

Currently, a hand full of primary intracranial neuroendocrine tumors (PINETs) have been reported in different anatomical locations such as the frontal convexity, sellar and hypothalamus, cavernous sinus, third ventricle, cerebellopontine angle, and the pineal gland.^[2,4,5]^ Clinically manifestations of PINETs are often a result of space-occurring effects and radiological evaluation often reveals no specific findings.^[2]^ Current standard treatment for PINETs includes complete tumor resection with adjuvant radiotherapy and chemotherapy.^[2,4–6]^ Herein, we present a very rare case of PINET that masqueraded as meningioma at the sphenoid ridge with metastasis to the spinal cord in a patient without a known history of extracranial NET at the time of diagnosis.

## 2. Case report

A 52-year-old male presented with 2 2-month history of headache and decreased vision in the left eye accompanied by inarticulation in speech for 1 month. He denied dizziness, voting, difficulty in swallowing, and hearing loss. He had no history of any chronic illness and no past history of hypertension and diabetes. On examination, his speech was less fluent which was suggestive of motor aphasia. Also, we notice gross memory decline. Ophthalmic examination revealed normal bilateral pupils which were sensitive to light reflex. Visual acuity was 3/6 in the left eye and 5/6 in the right eye. Examination of the other cranial nerves did not yield much. Power was 5/5 in upper and lower limbs and reflexes were normal. Kernig’s and Brudzinski’s signs were negative.

**Figure 1.**
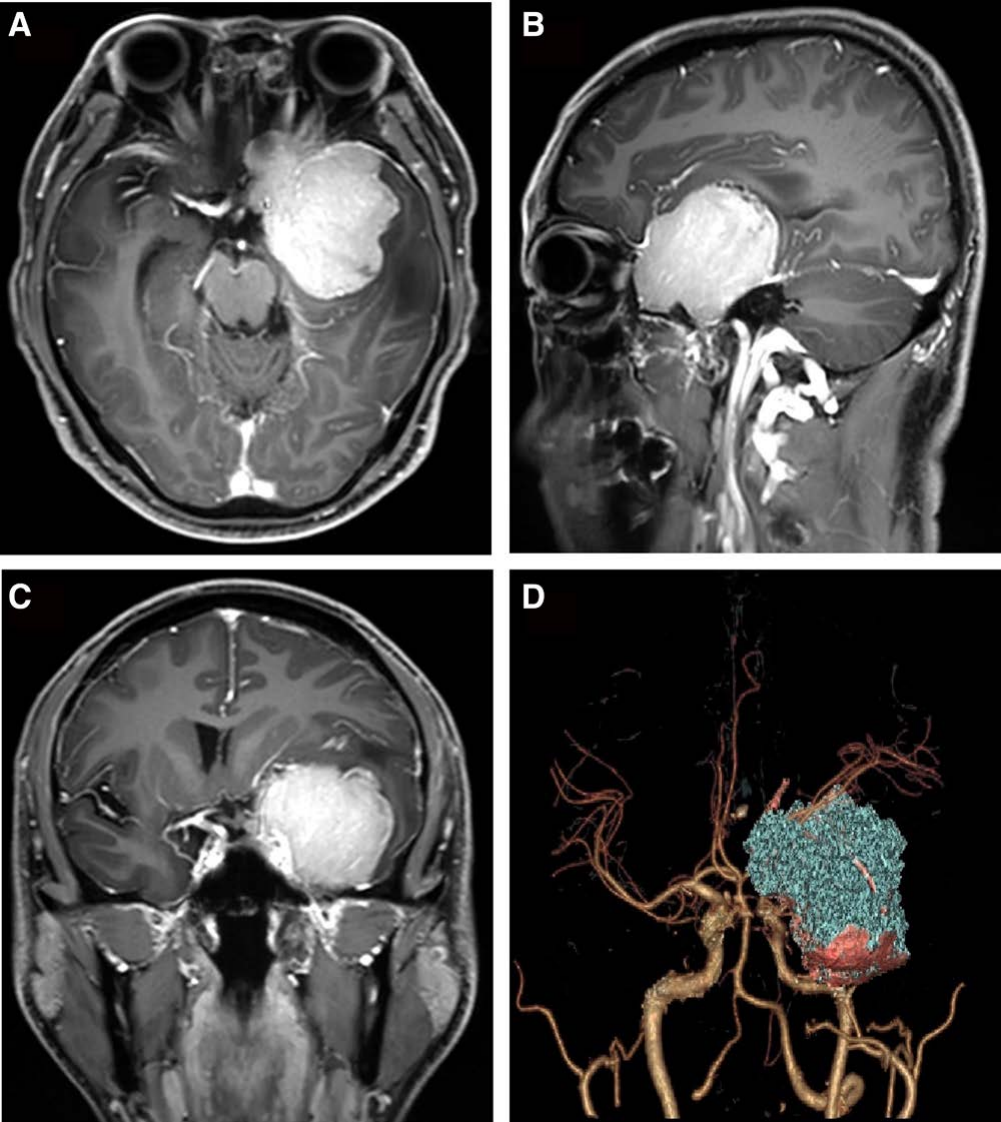
A preoperative MRI of the head showed a space-occupying lesion in the left middle cranial base or the sphenoid ridge. (A) Axial, (B) sagittal, (C) coronary. MRI = magnetic resonance imaging.

Routine laboratory investigations, chest X-ray, and electrocardiogram were normal. Magnetic resonance imaging (MRI) of the head showed a space-occupying lesion in the left middle cranial base or the sphenoid ridge (Fig. 1A–C). The lesion appears as an isointense on T1-weighted imaging, an iso- to high-intensity on T2-weighted imaging, and homogeneous enhancement on T1-weighted imaging with gadolinium contrast. Notably, computed tomography angiography of the head showed that the tumor was surrounded by the left internal carotid artery, the middle cerebral artery, and the optic nerve canal (Fig. 1D). Based on the radiological findings, preoperative diagnosis of left sphenoid ridge meningioma was established. Thus, the patient was scheduled for surgery.

Preoperatively, after satisfactory general anesthesia, the patient was placed in the supine position, the head of the bed was raised by 15°, and the patient head was tilted to the right side by 30°. A marking line of a curved incision was made and neuronavigation was used to determine the exact location and extent tumor resection. We accessed the tumor through an expanded left pterional approach. Intraoperatively, the tumor was solid with a rich blood supply. Also, the left internal carotid artery, middle cerebral artery, optic nerve, oculomotor nerve, and abducent nerve were compressed. The optic nerve, oculomotor nerve, and abducent nerve were intact and normal. The tumor resection was done in a piecemeal fashion. We attained total tumor resection. However, we observed cerebrospinal fluid leakage after suturing the dura, and artificial dura mater was used to successfully repair the dura defect. The bone flap was fixed with plates and screws.

**Figure 2.**
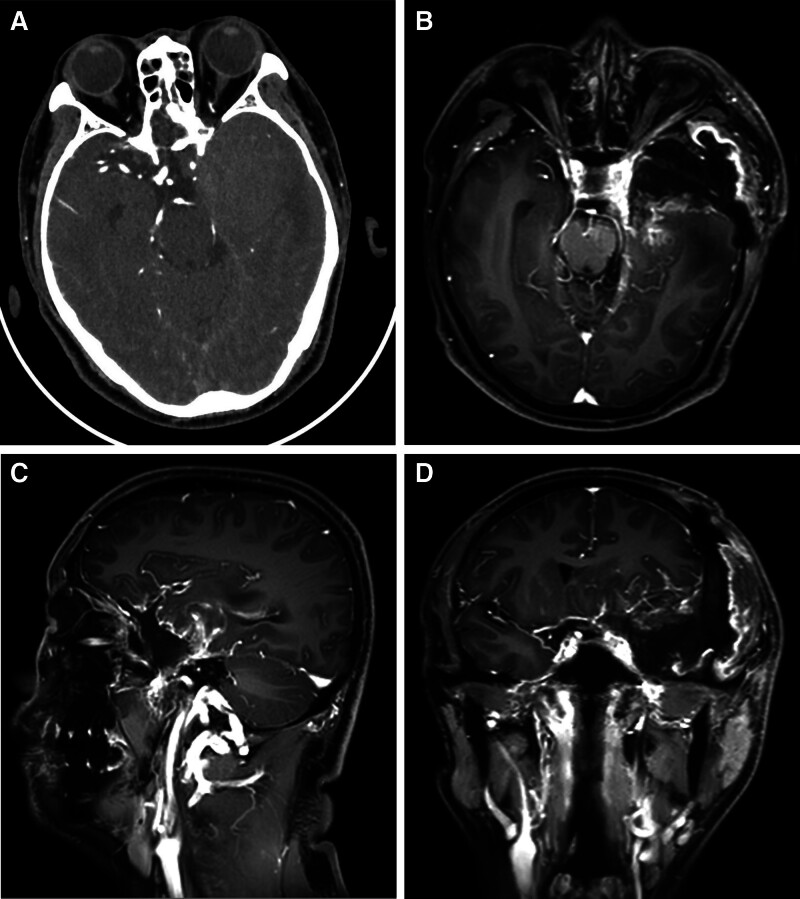
Postoperative CT scan and MRI show complete tumor resection. (A) CT scan, (B) axial MRI, (C) sagittal MRI, and (D) coronary MRI. CT = computed tomography, MRI = magnetic resonance imaging.

Postoperative CT scan (Fig. 2A) and MRI (Fig. 2C–D) showed complete tumor resection.

**Figure 3.**
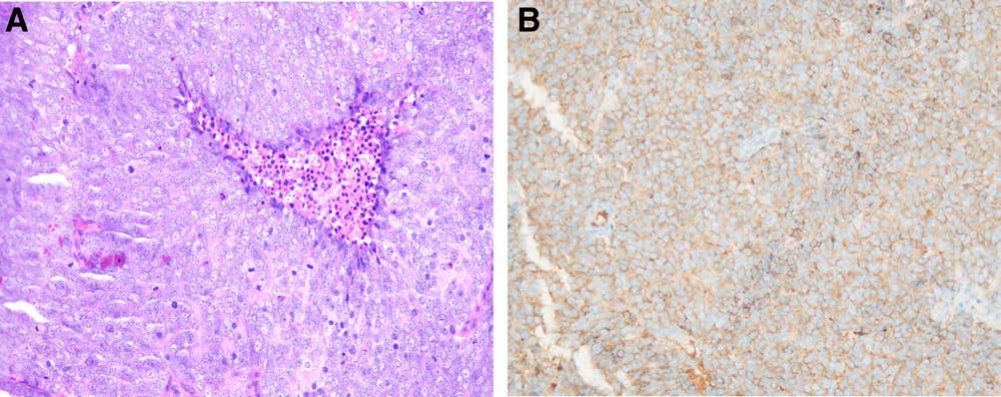
(A and B) Immunohistochemical images confirming that the tumor is NEC according to the World Health Organization classification. NEC = neuroendocrine carcinoma.

Also, postoperative pathological examination showed poorly differentiated lesion. Immunohistochemical (Fig. 3A, B) results showed positivity for cytokeratin (CK)-pan (spotty), epithelial membrane antigen (EMA) (a few), chromogranin A, synaptophysin, P53 (about 20%), retinoblastoma protein without reduction, and progesterone receptor about 5% with Ki-67 (MIB-1)-labeling index of about 85%. Based on the high Ki-67 labeling index above, PINET was established. It was a carcinoma according to the World Health Organization classification.

Also, immunohistochemical results showed negativity for Sry-related HMg-Box gene 10, CK5, 6, 7 and 20, thyroid transcription factor 1 or 8G7G3/1, glial fibrillary acidic protein (GFAP), caudal-type homeobox 2 (CDX2), special AT-rich sequence-binding protein 2 (SATB2), vimentin, NKX3.1, and >40% somatostatin receptor 2. Also, in situ hybridization results show negativity for EBER1/2-ISH. Thus, a definitive diagnosis of PINET was made. This diagnosis prompted us to thoroughly investigate the patient because of the multi-organ dissemination of this kind of tumor.

**Figure 4.**
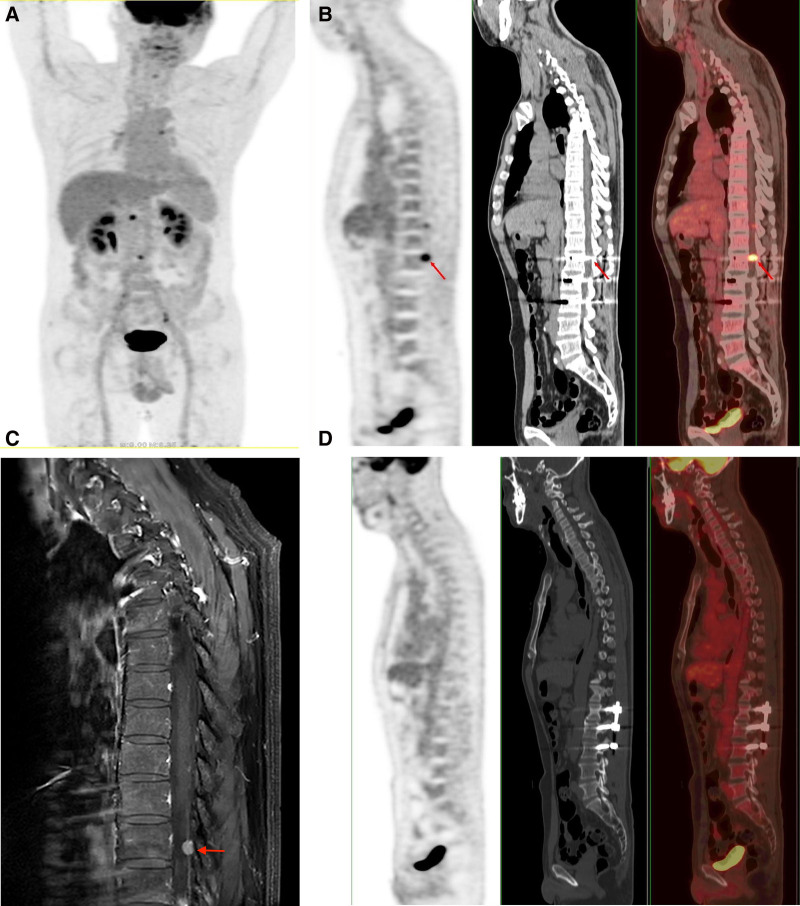
PET-CT scan images and MRI. (A) Fist postoperative PET-CT scan showing no primary lesions in other parts of the body. (B) Fist postoperative PET-CT scan showing a metastatic nodule in the spinal canal. Red arrow = metastatic nodule. (C) Fist postoperative MRI showing a metastatic nodule in the spinal canal. Red arrow = metastatic nodule. (D) Second postoperative PET-CT scan confirming total surgical resection of the spinal lesion and spinal fixations. CT = computed tomography, MRI = magnetic resonance imaging, PET = positron emission tomography.

Postoperative abdominal ultrasound showed no organomegaly’s and ascites. Also, positron emission tomography (PET)-CT scan of the chest and abdomen (Fig. 4A), gastroscopy did not show any primary lesions. However, PET-CT scan suggested a metastatic nodule in the spinal canal (Fig. 4B). MRI of the thoracolumbar spine (Fig. 4C) also showed a nodule in the thoracolumbar region which was a metastatic tumor. The metastatic lesion at thoracolumbar spine was surgically resected and spinal fixation was carried out to stabilize the spine (Fig. 4D). Interestingly, immunohistochemical evaluations revealed the same results as the lesions obtained for the sphenoid ridge (Fig. 3A, B).

Based on the earlier findings, the patient was further treated with radiation at the tumor bed and the spinal cord at a total dose of 54 Gy in 30 fractions, then 4 cycles of chemotherapy regimen comprising of etoposide 170 mg on day 1 to 3 and cisplatin 40 mg on day 1 to 3 at 3 weeks of intervals. Two years of follow-up revealed no recurrence of the tumor and he is currently well and going for his daily activities with no neurological deficits. However, we are still following the patient because of the nature of the tumor.

## 3. Discussion

The neuroendocrine system is made up of a complex architecture of cells that are able to trigger NETs at extracranial locations in body.^[1]^ These tumors are still very rare, and they constitute about 4% to 6% of all extracranial tumors.^[7,8]^ Notably, PINETs are even extremely rare^[2,4,5]^ and seem to be more analogous to non-metastatic NETs in other parts of the body with a 10-year overall survival rate of 47%.^[1,7]^ The brain metastatic form of NETs constitutes about 1.5% to 5% of all patients and in 70% of these patients, the bronchi or the lungs are the location of the primary tumor.^[1]^ Also, in case of brain metastases, associated lymph node metastases were detected in 75% patients, liver metastases were discovered in 50% of these patients whereas primary unknown NET constituted 13% of these tumors.^[1,9]^

Our case is much more interesting because the primary tumor masqueraded as meningioma at the sphenoid ridge with metastasis to the spinal cord in a patient without a known history of extracranial NET at the time of diagnosis. PINETs have been reported in different intracranial anatomical locations such as the frontal convexity, sellar and hypothalamus, cavernous sinus, third ventricle, cerebellopontine angle, and the pineal gland.^[2,4,5]^ Interestingly, our case is the first PINET in which the tumor originated from the sphenoid ridge. In the CNS, numerous epithelial cells as well as their progenitors may be differentiated in neuroendocrine cells which are capable of originating into PINET.^[10]^

A literature performed by Caro-Osorio et al^[5]^ revealed that the origin of PINETs was extra-axially in 6 of 11 patients. Also, they observed that the origin of the PINET was the pineal parenchyma in the 6 patients and the other inside of the third ventricle in the remaining patients.^[5]^ Furthermore, it was observed that extracranial NET usually metastasizes to the brain parenchyma through hematogenous dissemination.^[5]^ Thus, PINENs are mostly extra-axial lesions. We report for the first time that PINET is capable of metastasizing to the spinal cord. Notably, in majority of extracranial NET, metastases were observed in the liver, lungs, and bone.^[5]^ However, the association of other sites like the CNS is much rarer.^[1]^

PINET symptomatology occurs according to the brain region involved. In most cases, the patients presented with gradually worsening headaches, nausea and vomiting, dizziness, diplopia, progressive numbness of the face, and facial palsy.^[4,5,11]^ Also, the time of onset of symptoms to diagnosis ranged from 5 days to 6 years, with no association between onset as well as grade of tumor.^[5]^ The cardinal symptomatology in our patients was headache and decreased vision in the left eye accompanied by inarticulation in speech.

The imaging characteristics of PINETs are usually non-specific and the lesion is often mistaken for lesions like meningioma, glomus tumor, schwannoma, as well as metastasis.^[4,12]^ Thus, the diagnosis dilemma was not different in our case because, based on our radiological findings, preoperative diagnosis of left sphenoid ridge meningioma was established. The standard imaging workup for PINETs often includes CT scans MRI and fluorodexyglucose PET-CT scans.^[2]^ However, in our case, computed tomography angiography of the head was very rewarding because it showed that the tumor was surrounded by the left internal carotid artery, the middle cerebral artery, and the optic nerve canal. On MRI imaging, the lesion appears as an isointense on T1-weighted imaging, an iso- to high-intensity on T2-weighted imaging, and homogeneous enhancement on T1-weighted imaging with gadolinium contrast which is similar to the initial MRI report published in literature.^[2,5]^

It is worth noting that fluorodexyglucose PET-CT scans have excellent sensitivity in detecting high metabolic rate associated with high-grade NET.^[7,13]^ Notably, 100% of biopsy-proven high-grade NETs were detected with PET-CTs.^[7]^ In our case, additional imaging tests, such as CT scan, MRI, and ultrasonic imaging of the chest, abdomen, neck, and the genitourinary system showed no extracranial lesions in other organs. However, PET-CT scan and MRI of the thoracolumbar spine showed a nodule which was a metastatic tumor. Also, no lesions were detected in other organs, and the patient manifested no symptoms that could be related to tumor occurrence in other parts of the body.

Surgical resection of the lesion is the optimal treatment of PINETs.^[2,4–6]^ However, patients who receive all-inclusive treatment modalities such as full course of chemotherapy and/or radiotherapy have demonstrated to have longer tumor-free survival than patients who were treated with only surgery.^[6,14,15]^ In our patients, both the sphenoid ridge lesion and the metastatic lesion at thoracolumbar spine were surgically resected and spinal fixation was carried out to stabilize the spine. Notably, a combination of platinum-based chemotherapy such as cisplatin, ifosfamide, and etoposide have been advocated for high-grade NETs,^[6,7]^ while radiotherapy has been demonstrated to be effective in poorly differentiated PINENs.^[6,14]^ It was observed that other tumors that were not surgically resected became ominously necrosed after several cycles of adjuvant chemotherapy and radiotherapy.^[15]^

In our case, the patient was further treated with adjuvant radiation at the tumor bed and the spinal cord with adjuvant chemotherapy after complete tumor resection because of the poorly differentiated nature of the tumor. Notably, neuroendocrine cells are obviously associated with the coordination of neurotransmitter-induce synthesis as well as the expression of biologically active substances like active amines and peptidyl hormones, which allow NETs to maintain distinctive methods for identification.^[5,16]^ Histological evaluation often revealed tumor comprised of small round cells with uniform nuclei as well as scant cytoplasm, with nidulant or stripe-shaped distribution.^[14]^ Also, on electron micrographs, neuroendocrine granules were sometimes detected.^[14]^ Pathological examination of the resected tumor in our case showed poorly differentiated tumor.

In our case as well as initially reported cases, immunohistochemical showed positive for epithelial markers, like CK8, CK18, and chromogranin A, as well as for neuroendocrine markers, like synaptophysin, chromoplast-specific protein A, and NSE.^[5,14]^ However, initial reports revealed negativity for adrenocorticotropic hormone, carcinoembryonic antigen, thyroid transcription factor 1, and EMA and Ki-67-labeling index of less than 1 %.^[5,14]^ We observed a few positivity for EMA, P53, retinoblastoma protein, and progesterone receptor with Ki-67 (MIB-1) of about 85%. Interestingly, immunohistochemical evaluations revealed the same results as the lesions obtained for the sphenoid ridge. PINETs may exhibit histopathological heterogeneity as revealed by different stages of pathological progression or involvement of different locations.^[15]^

Based on pathological findings, NETs were classified into 5 types, such as Grade 1, Grade 2, NET with large cell or small cell, mixed adeno-NEC, as well as hyperplastic and neoplastic lesions according to the grading by the Ki-67 labeling index in the 2010 World Health Organization classification.^[1,3,17]^ Notably, NETs with carcinomas features had the worst prognosis, comparing to NETs with well-differentiated features, with overall survival of a few weeks and 7.5 years, respectively.^[5]^ In our case, 2 years follow-up revealed no recurrence of the tumor and he is currently well and going for his daily activities with no neurological deficits. Nevertheless, we are still following the patient because of the nature of the tumor.

## 4. Conclusion

PINET may be capable of metastasizing to spinal cord. Also, at the sphenoid ridge, the primary tumor may masquerade as meningioma. All-inclusive treatment modalities such as surgery and adjuvant chemotherapy and radiotherapy are the golden rule for PINET. We advocate that patient with PINET should be followed for a longer period of time because of the nature of the tumor.

## Author contributions

**Conceptualization:** Siyuan Pan, Shuide Chen, Seidu A. Richard, Zhigang Lan.

**Data curation:** Siyuan Pan, Shuide Chen, Seidu A. Richard, Zhigang Lan.

**Formal analysis:** Siyuan Pan, Shuide Chen, Seidu A. Richard, Zhigang Lan.

**Investigation:** Siyuan Pan, Shuide Chen, Seidu A. Richard, Zhigang Lan.

**Methodology:** Siyuan Pan, Shuide Chen, Seidu A Richard, Zhigang Lan.

**Writing—review & editing:** Siyuan Pan, Shuide Chen, Seidu A Richard, Zhigang Lan.

**Writing—original draft:** Seidu A. Richard.

**Funding acquisition:** Zhigang Lan.

**Supervision:** Zhigang Lan.
